# A valuable tool for the treatment of hepatocellular carcinoma

**DOI:** 10.1590/0100-3984.2023.56.5e2

**Published:** 2023

**Authors:** Thiago José Penachim

**Affiliations:** 1 Coordinator of the Interventional Radiology Department of the Hospital Vera Cruz de Campinas, and Coordinator of the Interventional Radiology Residency Program at the Hospital das Clínicas of the Universidade Estadual de Campinas (Unicamp), Campinas, SP, Brazil. thiagopenachim@gmail.com.

Hepatocellular carcinoma (HCC) is the sixth leading type of cancer and the third leading cause of cancer-related death worldwide, with the vast majority of cases (90%) occurring in individuals with cirrhosis^(^1^)^.

For patients diagnosed with HCC and cirrhosis, liver transplantation (LT) is considered the ideal treatment because it removes the cancer and promotes the cure of the chronic liver disease. However, this therapeutic option presents two challenges. The first is the disproportionality between the number of donors and the number of patients who need LT. The second is post-transplant tumor recurrence, which affects up to 20% of patients and for which treatment options are limited^(^2^)^. Therefore, careful selection of LT candidates is crucial to avoid injustices and futile procedures. Two fundamental strategies are employed to achieve that objective: the careful selection of patients who are good candidates for transplantation; and optimization of the management of the disease while those patients are on the transplant waiting list.

Patients are selected for LT on the basis of the Milan criteria-a single tumor ≤ 5 cm in diameter or up to three tumors ≤ 3 cm in diameter. Therefore, only those with early-stage HCC are selected. After LT, the five-year survival rate is approximately 70%, similar to that of transplant recipients without a prior diagnosis of HCC, and the five-year tumor recurrence rate is less than 15%^(^3^,^4^)^.

In Brazil, the time on the transplant waiting list varies from state to state and is generally long due to the shortage of donor organs. In some regions of the country, patients can wait from several months to a year for a donor liver. That raises concerns regarding the possibility of death prior to LT or removal from the waiting list secondary to HCC growth, which can make a patient ineligible according to the Milan criteria, approximately 40% of LT candidates becoming ineligible in that way within their first 12 months on the waiting list^(^5^)^.

Although their use is still controversial, locoregional treatment techniques such as percutaneous thermal ablation (by radiofrequency or microwave) and transarterial chemoembolization (TACE) are now in the toolbox of the interventional radiologist, being routinely applied at transplant centers to cure or inhibit the growth of HCC. This strategy, known as bridging therapy, is indicated when the time on the waiting list exceeds six months, and its use can avoid acquired ineligibility, thus improving LT efficacy and excluding patients with neoplasia that is more aggressive^(^6^)^.

The current issue of Radiologia Brasileira includes the important publication of a study entitled “Results of transarterial chemoembolization of hepatocellular carcinoma as a bridging therapy to liver transplantation”, in which the authors evaluated the degree of tumor necrosis in HCC after TACE and its impact on patient survival^(^7^)^. Total necrosis was achieved in approximately 65% of the HCCs submitted to TACE, with no significant differences in efficacy between conventional TACE (using an embolic agent + chemotherapy) and drug-eluting bead TACE (using an embolic agent loaded with chemotherapeutic agents). Survival was found to be better among the patients in whom total necrosis of the HCC was achieved that among those in whom only partial necrosis was achieved, although the difference did not reach statistical significance. One especially noteworthy finding of the study was the excellent correlation observed between total HCC necrosis and the results of imaging tests (computed tomography or magnetic resonance imaging), as evaluated in accordance with the Modified Response Evaluation Criteria in Solid Tumor guidelines^(^8^)^, a correlation between the two being observed in 80% of the patients.

The results of the study in focus^(^7^)^ not only have an impact on the bridging therapy for patients with early-stage HCC but also influence the rescue therapy (downstaging) for patients with intermediate-stage HCC, who initially do not meet the Milan criteria for inclusion on the transplant waiting list^(^9^)^. In addition, the data obtained are also valuable for patients who are ineligible for transplantation, in whom TACE can be used as a palliative treatment, with or without another type of locoregional treatment, such as thermal ablation, or even immunotherapy, as demonstrated in recent studies^(^10^,^11^)^.

The complexity of treating HCC cannot be ignored. The only way to obtain favorable results is with a complete, individualized approach, established through collaborative multidisciplinary discussion. Finally, given the wide range of therapeutic options available, most recently including yttrium-90 radioembolization, a high-cost technique with limited availability, TACE remains a valuable, economically effective option, quite far from being relegated to oblivion in the toolbox^(^12^)^.
